# Automated Perfusion Calculations vs. Visual Scoring of Collaterals and CBV-ASPECTS: Has the Machine Surpassed the Eye?

**DOI:** 10.1007/s00062-020-00974-3

**Published:** 2020-11-20

**Authors:** Marios-Nikos Psychogios, Peter B. Sporns, Johanna Ospel, Aristeidis H. Katsanos, Reza Kabiri, Fabian A. Flottmann, Bijoy K. Menon, Mackenzie Horn, David S. Liebeskind, Tristan Honda, Marc Ribo, Manuel Requena Ruiz, Christoph Kabbasch, Thorsten Lichtenstein, Christoph J. Maurer, Ansgar Berlis, Victoria Hellstern, Hans Henkes, Markus A. Möhlenbruch, Fatih Seker, Marielle S. Ernst, Jan Liman, Georgios Tsivgoulis, Alex Brehm

**Affiliations:** 1 Department of Neuroradiology, Clinic for Radiology & Nuclear Medicine, University Hospital Basel, Spitalstr. 21, 4031 Basel, Switzerland; 2 Department of Diagnostic and Interventional Neuroradiology, University Medical Center Hamburg-Eppendorf, Hamburg, Germany; 3 Department of Clinical Neurosciences, Radiology and Community Health Sciences, Cumming School of Medicine, University of Calgary, Calgary, Canada; 4 Department of Medicine (Neurology), McMaster University/Population Health Research Institute, Hamilton, Canada; 5 Second Department of Neurology, National and Kapodistrian University of Athens, Athens, Greece; 6 Department of Neurology, University of California, Los Angeles, USA; 7 Department of Neurology, Hospital Vall d’Hebron, Barcelona, Spain; 8 Department of Radiology, University Hospital Cologne, Cologne, Germany; 9 Department of Neuroradiology, University Hospital Augsburg, Augsburg, Germany; 10 Department of Neuroradiology, Klinikum Stuttgart, Stuttgart, Germany; 11 Department of Neuroradiology, University Hospital Heidelberg, Heidelberg, Germany; 12 Department of Neurology, University Hospital Göttingen, Göttingen, Germany; 13 Department of Neurology, The University of Tennessee Health Science Center, Memphis, TN USA

**Keywords:** Acute ischemic stroke, Perfusion imaging, Collaterals, Automated evaluation, Patient selection

## Abstract

**Purpose:**

Use of automated perfusion software has gained importance for imaging of stroke patients for mechanical thrombectomy (MT). We aim to compare four perfusion software packages: 1) with respect to their association with 3‑month functional outcome after successful reperfusion with MT in comparison to visual Cerebral Blood Volume - Alberta Stroke Program Early CT Score (CBV-ASPECTS) and collateral scoring and 2) with respect to their agreement in estimation of core and penumbra volume.

**Methods:**

This retrospective, multicenter cohort study (2015–2019) analyzed data from 8 centers. We included patients who were functionally independent before and underwent successful MT of the middle cerebral artery. Primary outcome measurements were the relationship of core and penumbra volume calculated by each software, qualitative assessment of collaterals and CBV-APECTS with 3‑month functional outcome and disability (modified Rankin scale >2). Quantitative differences between perfusion software measurements were also assessed.

**Results:**

A total of 215 patients (57% women, median age 77 years) from 8 centers fulfilled the inclusion criteria. Multivariable analyses showed a significant association of RAPID core (common odds ratio, cOR 1.02; *p* = 0.015), CBV-ASPECTS (cOR 0.78; *p* = 0.007) and collaterals (cOR 0.78; *p* = 0.001) with 3‑month functional outcome (shift analysis), while RAPID core (OR 1.02; *p* = 0.018), CBV-ASPECTS (OR 0.77; *p* = 0.024), collaterals (OR 0.78; *p* = 0.007) and OLEA core (OR 1.02; *p* = 0.029) were significantly associated with 3‑month functional disability. Mean differences on core estimates between VEOcore and RAPID were 13.4 ml, between syngo.via and RAPID 30.0 ml and between OLEA and RAPID −3.2 ml.

**Conclusion:**

Collateral scoring, CBV-ASPECTS and RAPID were independently associated with functional outcome at 90 days. Core and Penumbra estimates using automated software packages varied significantly and should therefore be used with caution.

**Electronic supplementary material:**

The online version of this article (10.1007/s00062-020-00974-3) contains supplementary material, which is available to authorized users.

## Introduction

Software packages that automatically postprocess computed tomographic perfusion (CTP) images and provide CTP-based core and penumbra estimates have been used in various randomized controlled mechanical thrombectomy (MT) trials for acute ischemic stroke patients due to a large vessel occlusion (LVO) [1, 2]. The underlying concept is that of an ischemic core of irreversibly damaged tissue, surrounded by an area of oligemic tissue, the penumbra [3, 4]. In theory, immediate reperfusion of the penumbra would save the affected tissue at risk, and a large infarct core or/and a small core-penumbra mismatch would indicate that there is little or no tissue that can be saved. Thus, the general approach in many comprehensive stroke centers is to decide in favor of MT when there is a small infarct core and a large mismatch, and to refrain from it when a large core and only a small or no mismatch is present on CTP maps [5].

While current guidelines suggest that perfusion imaging is not required within the first 6 h after symptom onset [6, 7], perfusion is a relevant tool for the selection of patients within 6–24 h of last time known well, because the two late window MT trials, DAWN and DEFUSE 3 [8, 9] relied on perfusion-derived core and penumbra estimates for patient selection, and this has translated into guideline recommendations [7–9]. Both DAWN and DEFUSE 3 used the RAPID^TM^ software (https://ischemaview.com/home) to generate perfusion-based core and penumbra volumes; however, various other perfusion packages from different vendors have been introduced and refined the last years, none of which were tested and validated in randomized trials. While ischemic core is defined on relative cerebral blood flow in most software, parameters to assess the penumbra vary across vendors and software packages [1, 3, 10]. Collateral flow status is a visually assessed biomarker that has been used in past MT trials [11] and a secondary analysis of the MR CLEAN trial supported the association of collaterals and treatment effect in stroke patients with LVO [6]. Due to the additional cost and time requirements related to automated perfusion analysis, physicians often visually assess perfusion imaging by applying the Alberta Stroke Program Early CT Score (ASPECTS) to the color-coded perfusion maps as a decision support tool in LVO patients [12] rather than relying on postprocessed threshold maps.

In this multicenter study, we therefore aimed to determine the value of four different commercially available automated perfusion software packages for estimation of infarct core and penumbral mismatch in patients presenting with LVO. Additionally, we evaluated the association of core and penumbral volumes derived from these perfusion software packages, an established collateral score and the cerebral blood volume (CBV-)ASPECTS with functional outcome 90 days after MT and with the extent of cerebral infarction 24 h following stroke symptom onset.

## Material and Methods

The data that support the findings of this study are available from the corresponding author on reasonable request.

### Study Design

Large vessel occlusion patients from 8 high-volume stroke centers across Europe, USA and Canada between January 2015 and June 2019 were retrospectively analyzed. The patients were derived from registries kept by the respective centers. All registries were approved by the respective local ethics committee. All data were anonymized prior to extraction. Inclusion criteria were a) functional independence prior to the index event (modified Rankin scale, mRS ≤2), b) successful reperfusion defined as modified thrombolysis in cerebral infarction (mTICI) score of 2b or better, c) time from perfusion scan to successful reperfusion <180 min and d) occlusion of the M1 or M2 segment of the middle cerebral artery (MCA). Exclusion criteria were a) missing prestroke mRS or 90 days mRS, and b) failure to calculate the ischemic core/mismatch volume or collaterals/CBV-ASPECTS due to technical reasons.

### Study Population

A total of 215 patients (122, 57% women) from 8 centers were evaluated. Out of the 215 patients, 33 (15%) patients were excluded from the final analysis due to technical failure of at least 1 perfusion software. Median age was 77 years (interquartile range, IQR 67–83 years), and 63 patients (29%) presented with a wake-up stroke. Median National Institute of Health Stroke Scale (NIHSS) at presentation was 14 (IQR 8–18) and the perfusion datasets were acquired on median 88 min (IQR 66–145 min) after symptom onset. A summary of the baseline characteristics is shown in Table 1.

**Table 1:** Baseline characteristics of the patient cohort

Variable	*N* = 182
*Age (median) (IQR) (years)*	77 (68–83)
*Female sex (n) (%)*	104 (57%)
*Wake-up stroke (n) (%)*	56 (31%)
*Baseline NIHSS score (median) (IQR)*	13 (8–18)
*Hypertension (n) (%)*	144 (79%)
*Diabetes (n) (%)*	47 (26%)
*Hyperlipidemia (n) (%)*	82 (45%)
*Smoking (n) (%)*	29 (16%)
*Obesity (n) (%)*	40 (22%)
*Time from onset to imaging (median) (IQR) (min)*	90 (68–185)
*Time from imaging to reperfusion (median) (IQR) (min)*	83 (60–101)
*Time from onset to reperfusion (median) (IQR) (min)*	198 (145–270)
*Left side occlusion (n) (%)*	93 (51%)
*Occluded segment of MCA (n) (%)*	
M1	138 (76%)
M2	44 (24%)
*Intravenous thrombolysis (n) (%)*	104 (57%)
*Tandem occlusion (n) (%)*	18 (10%)
*mTICI score post thrombectomy (n) (%)*	
mTICI 2b	62 (34%)
mTICI 2c	29 (16%)
mTICI 3	91 (50%)
*Number of passes (median) (IQR)*	1 (1–2)
*ASPECTS 24* *h after stroke (median) (IQR)*	8 (6–9)
*90-day mRS (median) (IQR)*	3 (1–5)
*90-day functional independence (mRS <3) (No.) (%)*	89 (49%)
*90-day mortality (n) (%)*	44 (24%)

*ASPECTS* Alberta Stroke Program Early Computed Tomography Score*, IQR* interquartile range, *MCA* middle cerebral artery, *mRS* modified Rankin scale, *mTICI* modified treatment in cerebral ischemia, *NIHSS* National Institutes of Health Stroke Scale

### Outcome Measures

Primary outcomes were the association of the core and penumbral estimates derived from the four perfusion software, collaterals and CBV-ASPECTS with 3‑month functional outcome (mRS score shift analysis) and with 3‑month functional disability (mRS score >2). Secondary outcomes included the mean differences of the ischemic core and penumbral volumes derived from three other perfusion software packages compared to the established RAPID software.

Demographic data, vascular risk factors, mTICI scores, time from symptom onset to imaging, imaging to reperfusion, groin to reperfusion and neurological scores were extracted from the databases. Image analysis was performed by an experienced neuroradiologist blinded to patient’s functional outcome at 3 months, whereas neurological status was assessed by stroke neurologists at hospital admission, hospital discharge, and on follow-up. Successful recanalization was defined as mTICI 2b, 2c or 3. Perfusion imaging was acquired on the following scanners: Somatom Definition AS+ (Siemens Healthcare Sector, Forchheim, Germany), Philips Brilliance 6 (Philips Healthcare, Best, The Netherlands), Philips Brilliance 64 (Philips Healthcare), Philips iCT 256 (Philips Healthcare), Somatom Definition AS (Siemens Healthcare Sector), Somatom Definition Flash (Siemens Healthcare Sector), Revolution CT (GE Healthcare, Chalfont St Giles, UK) and Discovery CT (GE Healthcare) using standard perfusion protocols [13]. The study was approved by the ethics committee of the University Hospital Basel, Basel, Switzerland, in accordance with the Declaration of Helsinki, with waiver of informed consent. Data were deidentified as previously described.

### Software Packages and Image Analysis

Four different software packages were used for the estimation of core and penumbra volume: RAPID v4.9.1.2 (iSchemaView Inc, Menlo Parc, CA, USA); VEOcore v1.1 (VEObrain GmbH, Freiburg, Germany); OLEA v3.0-SP18 (OLEA medical Inc., La Ciotat, France) and syngo.via VB30A_HF06 (Siemens Healthineers AG, Erlangen, Germany). We used the vendor prespecified standard settings and thresholds in all software packages. Postprocessing was centralized and standardized for all datasets after the different centers sent the raw data of the perfusion software to the core laboratory. The CBV maps were calculated with syngo.via using the built-in CT Neuroperfusion workflow. The color maps were not manually adapted. One experienced (>10-year experience after board certification) neuroradiologist evaluated the CBV maps using the ASPECTS according to the current methodology and additionally scored the maximum intensity projections of the CT angiography (CTA) using the 10-point Menon collateral score [14]. The CBV-ASPECTS and collateral scores were assessed independently and blinded to all clinical data.

### Statistical Analysis

First, we calculated the median with corresponding IQR of the provided core and penumbra volumes for every software packages. We used the non-parametric Friedman’s test to assess for potential differences in estimated core and penumbra volumes across the different software packages. The Conover test was then used for post hoc pairwise comparisons between these different software packages. Correlations between these various platform specific core/penumbra volumes were also tested using the nonparametric Spearman’s test.

We then calculated the mean pairwise differences and corresponding 95% confidence intervals (CI) for penumbra and core volume estimates as provided by different software packages and assessed the degree of agreement graphically with the use of Bland-Altman plots. We additionally evaluated in multivariable regression models the association of noncontrast CT (NCCT-)ASPECTS at 24 h, 3‑month functional outcome assessed with the mRS score, and 3‑month functional disability (mRS >2) with the CBV-ASPECTS, collateral score, and core and penumbra volumes provided by different software packages. All baseline characteristics that contributed to the corresponding outcome of interest in the initial univariable ordinal and binary logistic regression analyses at *p*-values <0.1 were included in the multivariable models as candidate variables. The final variables that were independently associated in the multivariable binary or ordinal logistic regression analyses with the outcomes of interest were selected using a two-sided alpha value <0.05.

Finally, we performed separate receiver operating characteristic (ROC) curve analyses on the prognostic utility of CBV-ASPECTS, collateral score and core volumes provided by different software packages for 3‑month functional disability. The individual areas under the ROC curves (AUC) were compared with the DeLong’s test.

All statistical analyses were conducted with the Stata Statistical Software Release 13 (StataCorp LP, College Station, TX, USA).

## Results

### Univariable and Multivariable Analyses of Clinical and Imaging Outcomes

**Fig. 1:**
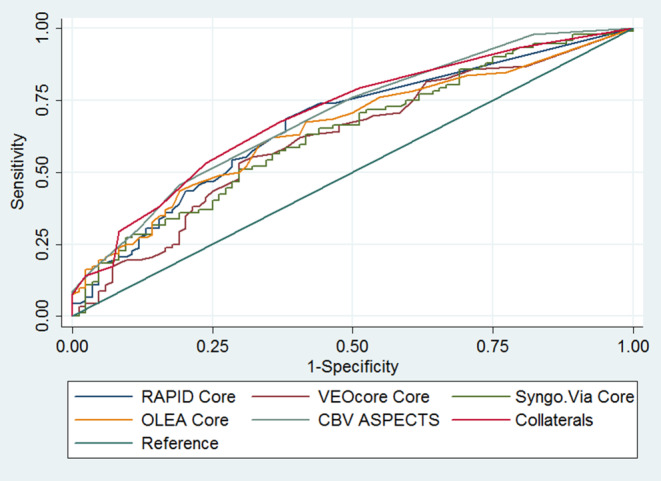
Receiver operating characteristic analyses for the prediction of disability (mRS >2) at 3 months for CBV-ASPECTS, collateral status and software core estimates

Results of the univariable logistic regression analyses on the association of baseline characteristics with the 3‑month mRS, with the 3‑month functional disability (mRS >2) and with the 24 h NCCT-ASPECTS can be found in eTables 1–3 of the online only supplements. Multivariable analyses showed a significant association of RAPID core measurements (common odds ratio, cOR per ml 1.02; 95% CI 1.01–1.03), CBV-ASPECTS (cOR per point 0.78; 95% CI 0.65–0.93) and collaterals (cOR per point 0.78; 95% CI 0.68–0.91) with the 3‑month functional outcome (Table 2). Similar findings were identified for the association of imaging parameters with the 3‑month functional disability as RAPID core (OR per ml 1.02; 95% CI 1.01–1.04), CBV-ASPECTS (OR per point 0.77; 95% CI 0.62–0.96), collaterals (OR per point 0.78; 95% CI 0.66–0.94) and OLEA core (OR per ml 1.02; 95%CI 1.00–1.05) emerged as independent predictors of 3‑month functional disability (Table 3). Regarding the association of baseline imaging parameters with the 24 h NCCT-ASPECTS, RAPID core, VEOcore core and OLEA core were all independently associated with the extent of infarction on follow-up imaging, while CBV-ASPECTS and collaterals showed the strongest association (eTable 4). The efigures 1–3 of the online-only supplements depict Bland-Altmann plots on the agreement of the various core measurements with core volumes measured with RAPID. Regarding the prediction of functional disability at day 90 poststroke, all imaging parameters had similar area under the curve values (*p*-value for DeLong’s test = 0.145; Table 4 and Fig. 1).

**Table 2:** Multivariable ordinal regression on the association of CBV-ASPECTS, collateral score and estimated core volume and penumbra volume with 90-day mRS, adjusted for baseline characteristics

Variable	Common OR (95%CI)	*p*-value
CBV-ASPECTS	0.78 (0.65, 0.93)	*0.007*
Collateral score	0.78 (0.68, 0.91)	*0.001*
RAPID Core volume (ml)	1.02 (1.01, 1.03)	*0.015*
RAPID Penumbra volume (ml)	1.00 (0.99, 1.01)	0.899
VEOcore Core volume (ml)	1.00 (0.99, 1.01)	0.456
VEOcore Penumbra volume (ml)	1.00 (0.99, 1.01)	0.586
syngo.via Core volume (ml)	1.00 (0.99, 1.01)	0.941
syngo.via Penumbra volume (ml)	1.01 (1.00, 1.02)	0.209
OLEA Core volume (ml)	1.01 (1.00, 1.03)	0.076
OLEA Penumbra volume (ml)	1.00 (1.00, 1.01)	0.369

All associations were adjusted for the variables: age, wake-up stroke, prestroke modified Rankin Scale (mRS), baseline National Institute of Health Stroke Scale (NIHSS), hypertension, diabetes, time from symptom onset to imaging, time from symptom onset to reperfusion, final modified Thrombolysis In Cerebral Infarction (mTICI), intravenous thrombolysis pretreatment; *CBV-ASPECTS* Cerebral Blood Volume Alberta Stroke Program Early CT Score, *p* Statistical significant

**Table 3:** Multivariable logistic regression analyses on the association of CBV-ASPECTS, collateral status and software core and penumbral parameters with 3‑month functional disability (mRS >2), adjusted for baseline characteristics

Variable	OR (95%CI)	*p*-value
CBV-ASPECTS	0.77 (0.62, 0.96)	*0.024*
Collateral score	0.78 (0.66, 0.94)	*0.007*
RAPID Core volume (ml)	1.02 (1.01, 1.04)	*0.018*
RAPID Penumbra volume (ml)	1.00 (0.99, 1.01)	0.412
VEOcore Core volume (ml)	1.00 (0.99, 1.00)	0.265
VEOcore Penumbra volume (ml)	1.00 (0.99, 1.01)	0.228
syngo.via Core volume (ml)	1.00 (0.99, 1.01)	0.283
syngo.via Penumbra volume (ml)	1.01 (1.00, 1.02)	0.080
OLEA Core volume (ml)	1.02 (1.00, 1.05)	*0.029*
OLEA Penumbra volume (ml)	1.00 (0.99, 1.01)	0.192

All associations were adjusted for the variables: age, pre-stroke modified Rankin Scale (mRS), baseline National Institute of Health Stroke Scale (NIHSS), hypertension, time from symptom onset to imaging, intravenous thrombolysis, final modified Thrombolysis In Cerebral Infarction (mTICI); *CBV-ASPECTS* Cerebral Blood Volume Alberta Stroke Program Early CT Score, *p* Statistical significant

**Table 4:** Prediction of disability (mRS >2) at 3 months with CBV-ASPECTS, collateral score and software core estimation

Variable	Area under the Curve	Standard Error	Asymptomatic Normal (95% Conf. Interval)
CBV-ASPECTS	0.6960	0.0385	0.6206–0.7715
Collateral score	0.7015	0.0388	0.6254–0.7776
RAPID Core volume (ml)	0.6672	0.0397	0.5894–0.7449
VEOcore Core volume (ml)	0.6181	0.0425	0.5348–0.7014
syngo.via Core volume (ml)	0.6356	0.0417	0.5539–0.7174
OLEA Core volume (ml)	0.6531	0.0411	0.5726–0.7337

Results are derived from ROC analysis; *CBV-ASPECTS* Cerebral Blood Volume Alberta Stroke Program Early CT Score

### Core and Penumbra Estimations with Different Perfusion Software

The comparison of core and penumbra estimations between the four software packages resulted in significant differences as shown in eTable 5. The mean difference with corresponding 95% CI on ischemic core estimates between VEOcore and RAPID was 13.4 ml (95% CI −9.8, 36.6), between syngo.via and RAPID 30 ml (95% CI 23.7, 36.4) and between OLEA and RAPID −3.2 ml (95% CI −6.1, −0.2) (Table 5). Positive mean differences indicated a larger mean core volume compared to RAPID, while negative mean differences indicated a smaller mean core volume compared to RAPID. Mean differences between software on penumbra volume estimates can be found in eTable 6. Spearman correlation coefficients between the different perfusion software regarding ischemic core and penumbra imaging estimation showed high variance and can be found in eTables 7 and 8.

**Table 5:** Mean pairwise differences (in ml) with corresponding 95% confidence intervals in brackets between the core volume estimates of the four software packages

	RAPID	VEOcore	syngo.via	OLEA
*RAPID*	–	13.4 (−9.8 to 36.6)	30.0 (23.7 to 36.4)	−3.2 (−6.1 to −0.2)
*VEOcore*	−13.4 (−36.6 to 9.8)	–	16.6 (−7.5 to 40.7)	−16.6 (−40.0 to 6.8)
*Syngo.Via*	−30.0 (−36.4 to −23.7)	−16.6 (−40.7 to 7.5)	–	−33.2 (−40.2 to −26.2)
*OLEA*	3.2 (0.2 to 6.1)	16.6 (−6.8 to 40.0)	33.2 (26.2 to 40.2)	–

Analysis performed with Bland-Altman plots

## Discussion

Our analysis indicates that visual assessment of a single-phase CTA collateral score or of CBV-ASPECTS are at least similar to automated perfusion evaluation in predicting functional outcome at 90 days poststroke. The model using single-phase CTA collaterals had the highest predictive power for functional disability at 90 days. Collaterals and CBV-ASPECTS were both independently associated with functional outcome and functional disability at 90 days. From the four software packages only the core estimate of RAPID was independently associated with functional outcome at 90 days.

After the publication of the DAWN and DEFUSE‑3 trials, automated estimations of ischemic core and penumbra volumes by RAPID became part of the guidelines for the selection of stroke patients eligible for MT in the late time window [7]; however, due to the costs of software packages, many hospitals prefer easily applicable selection tools, such as CBV-ASPECTS and collateral scoring in clinical routine. While CBV-ASPECTS has only been evaluated in smaller, retrospective cohorts [12] collateral scoring has already been validated in a large randomized controlled clinical trial for the time window of up to 12 h [11]. A post hoc analysis of patients randomized between 5.5–12 h after last time known well from the ESCAPE trial showed similar rates of favorable outcome as in the DAWN and DEFUSE‑3 trial [15], indicating that collaterals can be used for triage of stroke patients in the late time window. Furthermore, recently published work from Kim et al. and Almekhlafi et al. indicated that the treatment modifying effect of collaterals is of equal magnitude as perfusion-based paradigms in late time window patients, while potentially selecting more patients eligible for MT [16, 17]. Using only the DAWN and DEFUSE 3 criteria based on perfusion for the selection of late window patients might lead to the exclusion of patients who would benefit from MT. In a recently published sample 38% of the DAWN-ineligible and 41% of the DEFUSE 3‑ineligible patients had a good functional outcome at 90 days (mRS ≤2) [18]. As of now, optimal eligibility criteria for MT in the late window are not available; however, ongoing trials such as the TENSION (NCT03094715) trial might provide a better basis for evidence-based decisions with respect to MT in late-window stroke patients.

Another important aspect of our work was the comparison of the ischemic core and penumbra estimates of the different software packages. We showed that there are substantial differences in the estimates of the four software packages. Core volume estimates of OLEA and RAPID were similar, while we found relevant differences in the core volume estimation between VEOcore and RAPID (mean difference +13.4 ml) and syngo.via and RAPID (mean difference +30 ml). We also found relevant differences between RAPID and OLEA regarding the estimation of the penumbra volume (mean difference +41.9 ml). As for the mean differences between the core volume estimates of syngo.via/OLEA and RAPID our results are in line with smaller, previously published studies [19–21]. The VEOcore and penumbra volumes were not systematically evaluated so far. Consequently, our results pose a substantial limitation to the utilization of the DAWN and DEFUSE‑3 criteria on core volumes derived from VEOcore and syngo.via, as they might substantially overestimate the ischemic core volume and lead to the inappropriate disqualification of patients who are eligible for MT using the DAWN and DEFUSE‑3 criteria. On the other hand, the possible overestimation of the penumbra volume in OLEA would not lead to the disqualification of patients. In our study, the evaluation of core volumes by VEOcore and syngo.via was not associated with functional outcome or disability at 90 days. This indicates that the DAWN and DEFUSE imaging criteria based on RAPID cannot be applied to the alternative software packages.

While variability and interpretation of perfusion scans have benefited from automation, important technical challenges remain such as motion sensitivity, extra radiation and time delays with acquisition [16]. In our sample 5–7% (depending on the software) of the perfusion scans could not be processed, while we were able to evaluate all CTAs for collateral scoring. Another potential shortcoming of a perfusion-based paradigm is that the emerging one-stop management for stroke treatment (utilizing a flat-detector CT in the angiography suite) has not been evaluated in large patient cohorts with perfusion-based triage paradigms so far [22], while collateral scoring functions relatively well [23]. This deprives late time-window acute stroke patients of a potential time-saving effect of up to 40 min due to a reduction of in-hospital time delays [24, 25].

The predictive power for disability at 90 days of all examined paradigms was only modest with ROC values ranging between 0.62 and 0.70. This might be due to the fact that all of these paradigms do not consider the location of the ischemic lesion. As was pointed out by Ernst et al. in a post hoc analysis of the MR CLEAN dataset, the association between the infarct core volume and the outcome can be substantially strengthened by accounting for the mRS relevance of affected brain areas [26, 27].

One major strength of our study is that we used robust long-term outcome data, whereas other studies used the final infarct volume as a surrogate parameter for outcome [20, 21]; however, as was recently shown by Boers et al. in a pooled analysis from 7 randomized controlled trials, reduced final infarct volume only explained 12% of the treatment benefit in these trials [28], which was supported by a secondary analysis of the MR CLEAN data [29]. Since it was further shown that the association between final infarct volume and functional outcome is only moderate, it is questionable if this approach is suitable for evaluating the prognostic power of these software packages [30]. This notion is supported by our results as VEOcore was an independent predictor of the posttreatment ASPECTS but not of the functional outcome. Other strengths of our study include the high number of patients and the recruitment of patients from multiple high-volume stroke centers, which allows generalization of the results over different CT scanner systems.

Certain limitations of the present report need to be acknowledged. First, this was a retrospective analysis of prospectively collected data from different registries and thus our study is prone to selection bias. Second, the CBV-ASPECTS and collateral score were evaluated by only one experienced rater, limiting the extent to which our results can be generalized. Third, we included only patients with anterior circulation infarctions and were unable to evaluate the agreement or predictive value of the different neuroimaging software for the posterior circulation.

## Summary

Collateral scoring, CBV-ASPECTS and RAPID were independently associated with functional outcome at 90 days. Core and Penumbra estimates using automated software packages vary significantly and should therefore be used with caution.

## Caption Electronic Supplementary Material

The supplemental material contains additional etables and efigures for further clarification of the results as mentioned within the manuscript.
