# Supplementary material for: Automated Perfusion Calculations vs. Visual Scoring of Collaterals and CBV-ASPECTS: Has the Machine Surpassed the Eye?
Source: Clin Neuroradiol. 2020 Nov 20;31(2):499–506. doi: 10.1007/s00062-020-00974-3 (PMC8211603; doi:10.1007/s00062-020-00974-3)
Supplement: Supplementary file 1 — The supplemental material contains additional etables and efigures for further clarification of the results as mentioned within the manuscript. [file 62_2020_974_MOESM1_ESM.docx]

**SUPPLEMENTAL MATERIAL for the manuscript:**

“Automated perfusion calculations vs. visual scoring of collaterals and CBV-ASPECTS: Has the machine surpassed the eye?”

This supplemental material contains additional tables and figures for further clarification.

**Supplemental Tables:**

**eTable 1.** Univariable ordinal regression on the association of baseline characteristics with the 3-month functional outcome.

| **Variable** | **Common OR (95%CI)** | **p-value** |
| --- | --- | --- |
| Age | 1.05 (1.03, 1.06) | **<0.001** |
| Females | 1.23 (0.77, 1.98) | 0.390 |
| Wake-up stroke | 1.62 (0.95, 2.79) | **0.079** |
| Pre-stroke mRS | 2.47 (1.05, 5.80) | **0.038** |
| Baseline NIHSS | 1.09 (1.05, 1.14) | **<0.001** |
| Hypertension | 2.94 (1.63, 5.29) | **<0.001** |
| Diabetes | 1.68 (0.97, 2.29) | **0.066** |
| Hyperlipidemia | 0.92 (0.57, 1.49) | 0.745 |
| Smoking | 0.70 (0.34, 1.43) | 0.325 |
| Obesity | 0.82 (0.45, 1.51) | 0.534 |
| Onset-to-imaging time | 1.01 (1.01, 1.02) | **0.016** |
| Imaging-to-groin puncture time | 0.99 (0.99, 1.01) | 0.190 |
| Groin puncture to reperfusion time | 1.00 (0.99, 1.01) | 0.698 |
| Imaging to reperfusion time | 1.00 (0.99, 1.01) | 0.216 |
| Onset-to-groin puncture time | 1.00 (0.99, 1.01) | 0.237 |
| Onset-to-reperfusion time | 1.00 (0.99, 1.01) | **0.064** |
| Left side occlusion | 1.33 (0.82, 2.15) | 0.240 |
| Occluded vessel M1-MCA | 1.41 (0.82, 2.44) | 0.218 |
| IVT treatment | 0.52 (0.32, 0.85) | **0.009** |
| Tandem occlusion | 0.95 (0.43, 2.10) | 0.902 |
| Final mTICI | 0.58 (0.35, 0.96) | **0.033** |
| Number of passes | 0.99 (0.80, 1.21) | 0.909 |
| Collaterals | 0.76 (0.69, 0.84) | **<0.001** |
| CBV-ASPECTS | 0.71 (0.62, 0.81) | **<0.001** |
| RAPID Core (ml) | 1.02 (1.01, 1.03) | **<0.001** |
| RAPID Penumbra (ml) | 1.00 (0.99, 1.00) | 0.173 |
| VEOcore Core (ml) | 1.00 (0.99, 1.01) | 0.842 |
| VEOcore Penumbra (ml) | 1.00 (0.99, 1.01) | 0.192 |
| syngo.via Core (ml) | 1.00 (0.99, 1.01) | 0.179 |
| syngo.via Penumbra (ml) | 1.01 (0.99, 1.01) | **0.058** |
| OLEA Core (ml) | 1.02 (1.01, 1.03) | **0.002** |
| OLEA Penumbra (ml) | 1.01 (1.00, 1.01) | **0.044** |

ASPECTS Alberta Stroke Program Early CT Score, CBV Cerebral Blood Volume, IQR Inter-Quartile Range, IVT intravenous thrombolysis, M1-MCA M1 segment of the middle cerebral artery, mRS modified Rankin Scale, mTICI modified Treatment In Cerebral Ischemia, NIHSS National Institutes of Health Stroke Scale

**eTable 2.** Univariable logistic regression analyses on the association of baseline characteristics with the 3-month functional disability (mRS>2).

| **Variable** | **OR (95%CI)** | **p-value** |
| --- | --- | --- |
| Age | 1.05 (1.03, 1.08) | **<0.001** |
| Females | 1.20 (0.70, 2.06) | 0.514 |
| Wake-up stroke | 1.54 (0.82, 2,89) | 0.174 |
| Pre-stroke mRS | 5.01 (1.40, 17.98) | **0.013** |
| Baseline NIHSS | 1.11 (1.06, 1.16) | **<0.001** |
| Hypertension | 2.98 (1.48, 6.00) | **0.002** |
| Diabetes | 1.45 (0.77, 2.73) | 0.247 |
| Hyperlipidemia | 0.94 (0.54, 1.61) | 0.813 |
| Smoking | 0.78 (0.36, 1.71) | 0.547 |
| Obesity | 1.01 (0.50, 2.04) | 0.973 |
| Onset-to-imaging time | 1.00 (0.99, 1.01) | **0.078** |
| Imaging-to-groin puncture time | 0.99 (0.98, 1.00) | 0.182 |
| Groin puncture to reperfusion time | 1.00 (0.99, 1.01) | 0.977 |
| Imaging to reperfusion time | 1.00 (0.99, 1.01) | 0.427 |
| Onset-to-groin puncture time | 1.00 (0.99, 1.01) | 0.345 |
| Onset-to-reperfusion time | 1.00 (0.99, 1.01) | 0.166 |
| Left side occlusion | 1.40 (0.81, 2.39) | 0.244 |
| Occluded vessel M1-MCA | 1.57 (0.84, 2.92) | 0.158 |
| IVT treatment | 0.61 (0.35, 1.06) | **0.083** |
| Tandem occlusion | 0.86 (0.35, 2.13) | 0.749 |
| Final mTICI | 0.51 (0.29, 0.91) | **0.024** |
| Number of passes | 0.95 (0.74, 1.22) | 0.676 |
| Collaterals | 0.75 (0.65, 0.85) | **<0.001** |
| CBV-ASPECTS | 0.69 (0.58, 0.81) | **<0.001** |
| RAPID Core (ml) | 1.02 (1.01, 1.03) | **0.001** |
| RAPID Penumbra (ml) | 1.00 (0.99, 1.00) | 0.774 |
| VEOcore Core (ml) | 1.00 (0.99, 1.01) | 0.556 |
| VEOcore Penumbra (ml) | 1.00 (0.99, 1.01) | 0.167 |
| syngo.via Core (ml) | 1.01 (0.99, 1.01) | **0.075** |
| syngo.via Penumbra (ml) | 1.01 (1.00, 1.02) | **0.050** |
| OLEA Core (ml) | 1.02 (1.01, 1.04) | **0.005** |
| OLEA Mismatch (ml) | 1.00 (1.00, 1.01) | **0.040** |

ASPECTS Alberta Stroke Program Early CT Score, CBV Cerebral Blood Volume, IQR Inter-Quartile Range, IVT intravenous thrombolysis, M1-MCA M1 segment of the middle cerebral artery, mRS modified Rankin Scale, mTICI modified Treatment In Cerebral Ischemia, NIHSS National Institutes of Health Stroke Scale

**eTable 3.** Univariable ordinal regression on the association of baseline characteristics with the NCCT-ASPECTS at 24 hours.

| **Variable** | **Common OR (95%CI)** | **p-value** |
| --- | --- | --- |
| Age | 0.98 (0.96, 0.99) | **0.007** |
| Females | 0.69 (0.42, 1.15) | 0.157 |
| Wake-up stroke | 0.93 (0.53, 1.63) | 0.793 |
| Pre-stroke mRS | 1.23 (0.48, 3.18) | 0.657 |
| Baseline NIHSS | 0.92 (0.88, 0.96) | **<0.001** |
| Hypertension | 0.58 (0.32, 1.07) | **0.083** |
| Diabetes | 0.73 (0.40, 1.32) | 0.300 |
| Hyperlipidemia | 1.24 (0.74, 2.06) | 0.413 |
| Smoking | 1.08 (0.51, 2.30) | 0.835 |
| Obesity | 0.88 (0.47, 1.64) | 0.685 |
| Onset-to-imaging time | 0.99 (0.98, 0.99) | **<0.001** |
| Imaging-to-groin puncture time | 1.00 (0.99, 1.01) | **0.092** |
| Groin puncture to reperfusion time | 1.00 (0.99, 1.01) | 0.384 |
| Imaging to reperfusion time | 1.00 (0.99, 1.01) | 0.460 |
| Onset-to-groin puncture time | 0.99 (0.98, 0.99) | **0.020** |
| Onset-to-reperfusion time | 0.99 (0.98, 0.99) | **<0.001** |
| Left side occlusion | 0.68 (0.41, 1.12) | 0.131 |
| Occluded vessel M1-MCA | 0.54 (0.31, 0.95) | **0.034** |
| IVT treatment | 1.17 (0.70, 1.96) | 0.542 |
| Tandem occlusion | 0.64 (0.29, 1.39) | 0.259 |
| Final mTICI | 1.84 (1.06, 3.19) | **0.030** |
| Number of passes | 0.93 (0.74, 1.17) | 0.557 |
| Collaterals | 1.32 (1.18, 1.47) | **<0.001** |
| CBV-ASPECTS | 1.53 (1.33, 1.76) | **<0.001** |
| RAPID Core (ml) | 0.98 (0.97, 0.99) | **<0.001** |
| RAPID Penumbra (ml) | 1.00 (0.99, 1.01) | 0.487 |
| VEOcore Core (ml) | 0.98 (0.97, 0.99) | **<0.001** |
| VEOcore Penumbra (ml) | 1.00 (0.99, 1.01) | 0.862 |
| syngo.via Core (ml) | 1.00 (0.99, 1.01) | 0.255 |
| syngo.via Penumbra (ml) | 1.00 (0.99, 1.01) | 0.269 |
| OLEA Core (ml) | 0.97 (0.96, 0.98) | **<0.001** |
| OLEA Penumbra (ml) | 1.00 (0.99, 1.01) | 0.699 |

ASPECTS Alberta Stroke Program Early CT Score, CBV Cerebral Blood Volume, IQR Inter-Quartile Range, IVT intravenous thrombolysis, M1-MCA M1 segment of the middle cerebral artery, mRS modified Rankin Scale, mTICI modified Treatment In Cerebral Ischemia, NIHSS National Institutes of Health Stroke Scale

**eTable 4.** Multivariable ordinal regression on the association of CBV-ASPECTS, collateral score and estimated core volume and penumbra volume with NCCT-ASPECTS 24h adjusted for baseline characteristics

| **Variable** | **Common OR (95%CI)** | **p-value** |
| --- | --- | --- |
| CBV-ASPECTS | 1.62 (1.32, 1.98) | **<0.001** |
| Collateral score | 1.31 (1.13, 1.51) | **<0.001** |
| RAPID Core volume (ml) | 0.98 (0.96, 0.99) | **0.001** |
| RAPID Penumbra volume (ml) | 1.00 (0.99, 1.01) | 0.743 |
| VEOcore Core volume (ml) | 0.98 (0.97, 0.99) | **0.024** |
| VEOcore Penumbra volume (ml) | 1.00 (0.99, 1.01) | 0.694 |
| syngo.via Core volume (ml) | 1.00 (0.99, 1.01) | 0.354 |
| syngo.via Penumbra volume (ml) | 1.00 (0.99, 1.01) | 0.799 |
| OLEA Core volume (ml)OLEA Core volume (ml) | 0.97 (0.96, 0.99) | **0.001** |
| OLEA Penumbra volume (ml) | 1.00 (0.99, 1.01) | 0.968 |

All models were adjusted for the variables: age, baseline NIHSS score, hypertension, time from symptom onset to imaging, imaging-to-groin puncture time, onset-to-groin puncture time, onset-to-reperfusion time, mTICI

**eTable 5.** Comparison of the estimated core and penumbra volumes in ml of the four software packages

|  | **Core** | **Penumbra** |
| --- | --- | --- |
| RAPID (median) (IQR) | 7 (0-27) | 71 (44-104) |
| VEOcore (median) (IQR) | 13 (3-28) | 78 (47-110) |
| syngo.via (median) (IQR) | 36 (23-58) | 75 (48-100) |
| OLEA (median) (IQR) | 8 (2-22) | 98 (61-138) |
|  | **P<0.001** | **P<0.001** |

Analysis performed with the Friedman’s test and differences between groups were assessed with the Conover test

**eTable 6.** Mean differences in ml with corresponding 95% confidence intervals between the penumbral volume estimates of the four software solutions

|  | **RAPID** | **VEOcore** | **syngo.via** | **OLEA** |
| --- | --- | --- | --- | --- |
| **RAPID** | - | 4.4  (-6.3 to 15.0) | -3.0  (-13.5 to 7.5) | 41.9  (25.5 to 58.2) |
| **VEOcore** | -4.4  (-15.0 to 6.3) | - | -7.4  (-16.8 to 2.1) | 37.5  (21.8 to 53.2) |
| **syngo.via** | 3.0  (-7.5 to 13.5) | 7.4  (-2.1 to 16.8) | - | 44.8  (28.8 to 60.9) |
| **OLEA** | -41.9  (-58.2 to -25.5) | -37.5  (-53.2 to -21.8) | -44.8  (-60.9 to -28.8) | - |

Analysis performed with Bland-Altman plots

**eTable 7.** Spearman correlation coefficients between software on ischemic core estimates.

|  | **RAPID** | **VEOcore** | **syngo.via** | **OLEA** |
| --- | --- | --- | --- | --- |
| **RAPID** | - | rho=0.808  p<0.001 | rho=0.623  p<0.001 | rho=0.605  p<0.001 |
| **VEOcore** | rho=0.808  p<0.001 | - | rho=0.485  p<0.001 | rho=0.630  p<0.001 |
| **syngo.via** | rho=0.623  p<0.001 | rho=0.485  p<0.001 | - | rho=0.338  p<0.001 |
| **OLEA** | rho=0.605  p<0.001 | rho=0.630  p<0.001 | rho=0.338  p<0.001 | - |

**eTable 8.** Spearman correlation coefficients between software on penumbral volume estimates.

|  | **RAPID** | **VEOcore** | **syngo.via** | **OLEA** |
| --- | --- | --- | --- | --- |
| **RAPID** | - | rho=0.711  p<0.001 | rho=0.339  p<0.001 | rho=0.594  p<0.001 |
| **VEOcore** | rho=0.711  p<0.001 | - | rho=0.284  p<0.001 | rho=0.598  p<0.001 |
| **syngo.via** | rho=0.339  p<0.001 | rho=0.284  p<0.001 | - | rho=0.242  p=0.001 |
| **OLEA** | rho=0.594  p<0.001 | rho=0.598  p<0.001 | rho=0.242  p=0.001 | - |

**Supplemental Figures:**


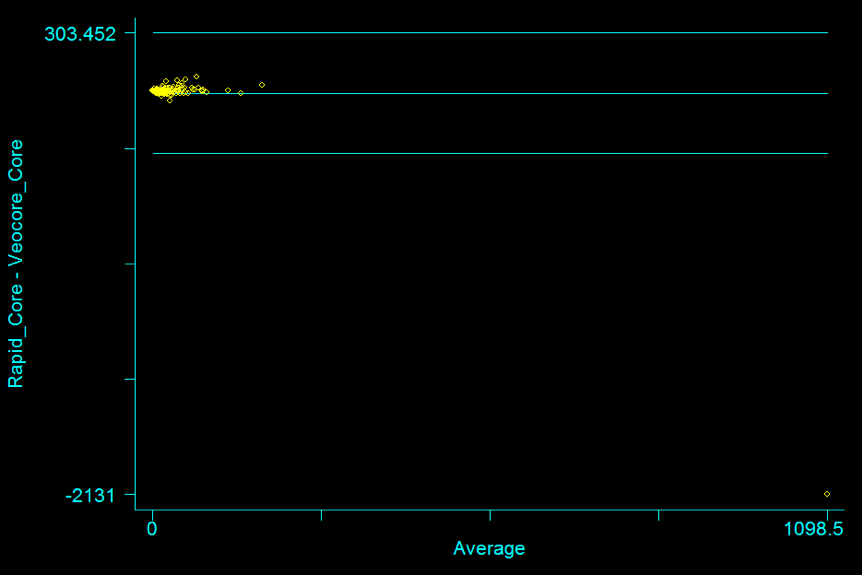


**eFigure 1.** Bland-Altman plot on the comparison of RAPID core with VEOcore core


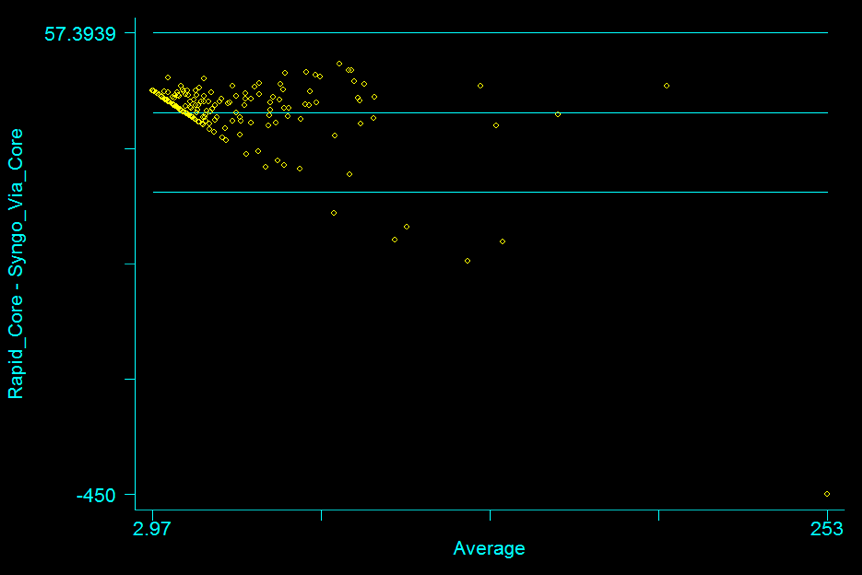


**eFigure 2.** Bland-Altman plot on the comparison of RAPID core with syngo.via core


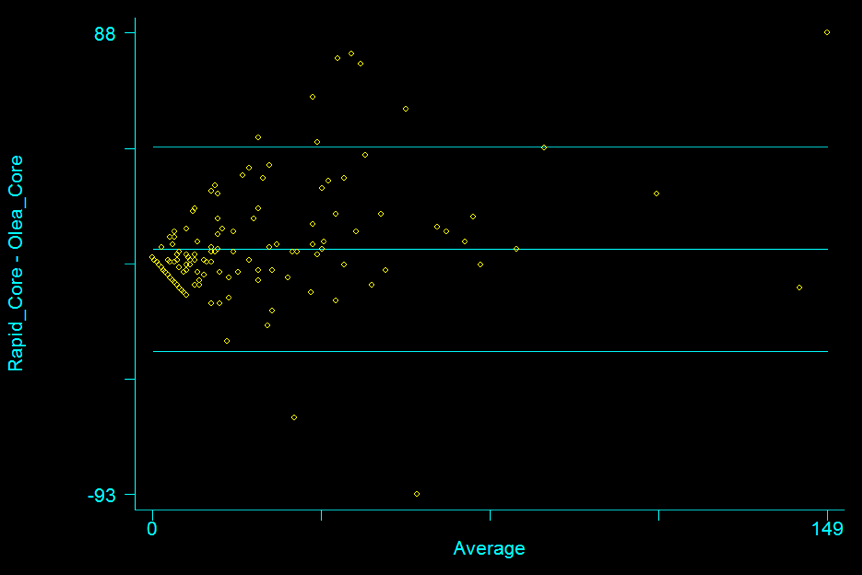


**eFigure 3.** Bland-Altman plot on the comparison of RAPID core with OLEA core
